# Neutrophil-to-Lymphocyte, Platelet-to-Lymphocyte Ratios, and Systemic Immune-Inflammation Index as Potential Biomarkers of Chronic Inflammation in Patients with Newly Diagnosed Acromegaly: A Single-Centre Study

**DOI:** 10.3390/jcm10173997

**Published:** 2021-09-03

**Authors:** Joanna Szydełko, Magdalena Szydełko-Gorzkowicz, Beata Matyjaszek-Matuszek

**Affiliations:** 1 Department of Endocrinology, Diabetology and Metabolic Diseases, Medical University of Lublin, Jaczewskiego 8, 20-090 Lublin, Poland; bmm@2com.pl; 2 Department of Obstetrics and Perinatology, Independent Public Clinical Hospital No. 4 in Lublin, Jaczewskiego 8, 20-954 Lublin, Poland

**Keywords:** neutrophil-to-lymphocyte ratio, platelet-to-lymphocyte ratio, systemic immune-inflammation index, acromegaly, non-functioning pituitary adenomas

## Abstract

Acromegaly is a rare disease caused by overproduction of growth hormone (GH) by a pituitary adenoma, and consequently increased insulin-like growth factor 1 (IGF-1) concentration. The GH/IGF-1 axis and immune cells interactions are hypothesized to be involved in subclinical inflammation. This retrospective study aimed to investigate the differences in neutrophil-to-lymphocyte (NLR), platelet-to-lymphocyte (PLR) ratios, and systemic immune-inflammation index (SII) in GH-secreting adenomas compared with non-functioning pituitary adenomas (NFPAs) concerning clinical and radiological findings. After evaluation of 665 patients with pituitary tumors, 62 individuals with newly diagnosed acromegaly and 134 with NFPAs were enrolled in the analysis. The control group consisted of 120 healthy individuals. Fifty-eight patients with acromegaly were re-evaluated after medical or surgical therapies. NLR, PLR, SII values, and neutrophil count were significantly higher (*p* ≤ 0.001), whereas lymphocyte count was lower in acromegaly than in NFPAs (*p* = 0.001). No significant differences between NFPAs and controls were observed in analyzed ratios. Higher preoperative NLR, PLR, SII values were found in patients who failed to achieve a cure with surgery (*p* < 0.05). Although NLR, PLR, and SII values were significantly higher in acromegaly, these indices cannot be used to discriminate GH-secreting pituitary tumors from NFPAs. Treatment of acromegaly decreased the value of NLR and SII, but it requires further studies to consolidate the real clinical role of these inflammation-related ratios.

## 1. Introduction

Acromegaly is a rare chronic disease caused by excessive production of growth hormone (GH), mostly by a pituitary adenoma, and subsequent insulin-like growth factor 1 (IGF-1) excess [1]. The prevalence of acromegaly is estimated at only 50–70 cases per million inhabitants and it does not vary with gender [2,3]. GH-secreting pituitary tumors are characterized by a benign nature and follow an indolent course [1]. Initially, the clinical features of acromegaly may be very subtle, which most likely results in a prolonged diagnostic delay of an average of 5 to 10 years after the onset of first signs and symptoms [4]. It is well established that long-lasting GH/IGF-1 axis hypersecretion leads to a number of systemic complications, especially increased risk of cardiovascular diseases that reduce the patient’s quality of life and survival [5].

Recent studies have proved that local and systemic inflammation may modulate and promote several tumorigenic processes, such as tumor cell proliferation, migration, invasion, angiogenesis, and immune cell chemoattraction in different cancers [6,7]. Findings from clinical and basic research studies have strongly indicated the importance of supraphysiological doses of GH and IGF-1 in initiating the inflammatory process via cytokine production by immune cells [5,8]. T-cells, B-cells, and monocytes are concerned to play a crucial role in the bi-directional communication network between the endocrine and immune systems in patients with acromegaly [9,10].

As cost-effective, easily-accessible, and commonly used indices, complete blood count (CBC)-derived parameters have been a subject of high interest among researchers over the past decades [11,12,13]. It is hypothesized that increased absolute neutrophil count is caused by the hypersecretion of myeloid growth factors by tumor cells and/or neoplasm-related hypercytokinaemia. Conversely, it is well-known that absolute lymphocyte count is reduced in the natural course of neoplasms and it may be associated with poor clinical outcomes [14,15,16]. Several studies have proved the neutrophil-to-lymphocyte ratio (NLR), lymphocyte-to-monocyte ratio (LMR), platelet-to-lymphocyte ratio (PLR), mean platelet volume-to-platelet ratio (MPV/PLT), and systemic immune-inflammation index (SII) as novel biomarkers of chronic subclinical inflammation in the thyroid, adrenal gland, and neuroendocrine tumors [14,17,18,19,20]. Moreover, these ratios have been proposed as potential indicators to differentiate malignant from benign neoplasms in the preoperative period, and predict the prognosis and response to treatment [21,22,23,24,25,26,27]. Some authors proved that preoperative NLR and PLR values may provide effective guidance about long-term outcomes, including the recurrence rate and progression-free survival in craniopharyngiomas and meningiomas [28,29,30,31].

However, little is known about hematological parameters in patients with acromegaly and NFPAs. There are only single studies evaluating the relationship between these direct and indirect blood cell-associated inflammatory indices and pituitary tumors [32,33,34,35,36,37].

Therefore, the present study aimed to assess the value of selected CBC-derived parameters, such as the NLR, LMR, PLR, MPV/PLT, and SII in GH-secreting adenomas as compared to non-functioning pituitary adenomas (NFPAs) concerning clinical and radiological findings.

## 2. Materials and Methods

### 2.1. Statement of Ethics

The study protocol was approved on 27 February 2020 by the Bioethics Committee of the Medical University of Lublin, Poland (approval No. KE-0254/43/2020). Due to the retrospective nature of the study, informed consent was not required, and patients’ data were used anonymously. The research was conducted in accordance with Good Clinical Practice (Declaration of Helsinki of 1975, revised in 2013).

### 2.2. Study Design and Patients

**Figure 1:**
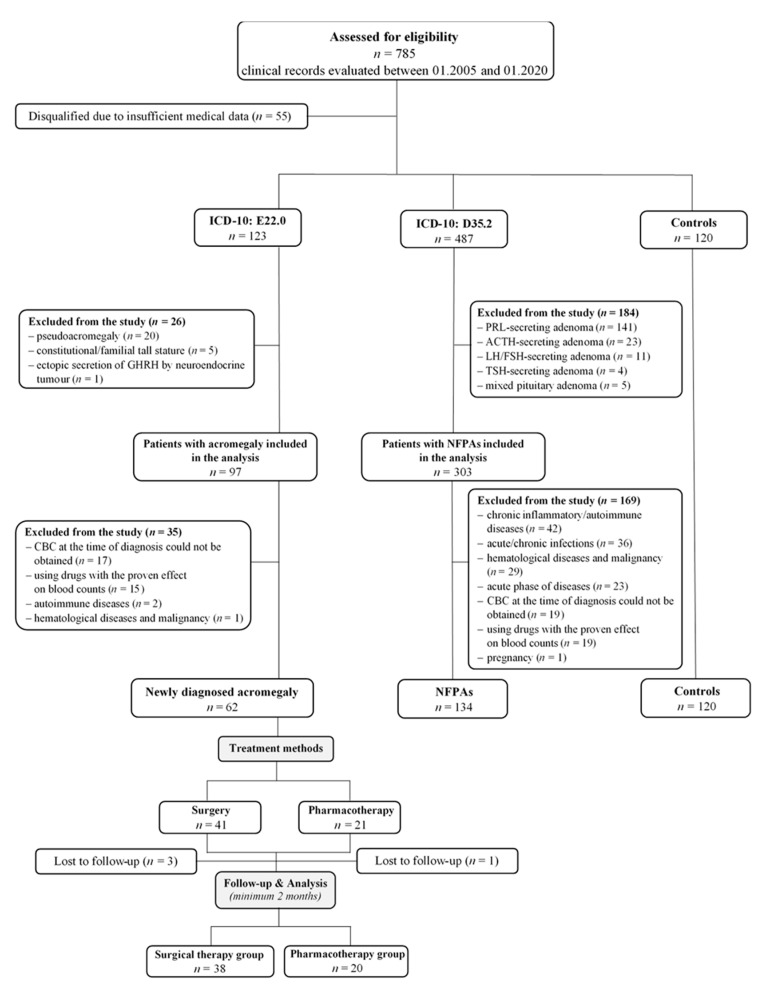
Flow chart of the study. ICD-10—International Classification of Diseases revision 10; CBC—complete blood count; NFPA—non-functioning pituitary adenoma; GHRH—growth hormone-releasing hormone; PRL—prolactin; ACTH—adrenocorticotropic hormone; LH—luteinizing hormone; FSH—follicle-stimulating hormone; TSH—thyroid-stimulating hormone.

The research was designed as a retrospective, cross-sectional, and observational, single-center study. Patients’ data were obtained from paper-based and/or electronic charts. The medical records of 785 patients identified with codes E22.0 and D35.2 according to the International Classification of Diseases revision 10 (ICD-10), hospitalized in the Department of Endocrinology, Diabetology and Metabolic Diseases at the Medical University of Lublin, Poland, during the period between 1 January 2005 and 31 January 2020 were reviewed. The diagnosis of acromegaly was based on the presence of characteristic clinical features, increased secretion of IGF-1 adjusted for age and sex, and inability to suppress the GH level < 1 ng/mL in an oral glucose tolerance test (OGTT) after administration of 75 g of glucose according to the Endocrine Society Clinical Practice Guideline [38]. In patients with contraindications to performing OGTT, e.g., previously diagnosed diabetes mellitus, the GH concentration was tested several times (every 30 min for 2–3 h) and the average level < 2.5 ng/mL was allowed to exclude acromegaly [3,39]. Following the biochemical diagnosis of acromegaly, magnetic resonance imaging (MRI) with contrast enhancement of the sella turcica region was performed to visualize tumor size and appearance, as well as parasellar extent [38,40]. The NFPA was defined as a hypointense or isointense tumor on T1-weighted images on the MRI and the absence of clinical and biochemical evidence of hormonal hypersecretion [41]. The control group consisted of 120 randomly selected, healthy individuals without disturbances of the hypothalamic-pituitary system and other diseases affecting CBC-derived parameters. The exclusion criteria for the study and control groups were as follows: acute and chronic infection, autoimmune/inflammatory systemic diseases, intracranial surgery or trauma history within the central nervous system during the past 12 months, acute phase of diseases including pituitary apoplexy, other cerebrovascular events, myocardial infarction, severe hepatic or renal dysfunction, using systemic drugs with the proven effect on blood counts, adrenocorticotropic axis insufficiency during replacement therapy (at diagnosis or after surgery), pregnancy, past or present malignancy history and hematological disorders, constitutional/familial tall stature and pseudoacromegaly, ectopic secretion of growth hormone-releasing hormone (GHRH) or GH by a neuroendocrine tumor, other hormone-secreting pituitary adenomas. Moreover, patients with incomplete medical documentation or those who did not have a CBC test at the time of diagnosis were also excluded from the study. After evaluation for eligibility, 62 patients with acromegaly (Group 1), 134 with NFPAs (Group 2), and 120 healthy individuals (Group 3) were enrolled in the analysis. The inclusion and exclusion criteria, as well as the inclusion chart, are presented in Figure 1.

All patients with acromegaly were divided according to the treatment method. Forty one of them underwent transsphenoidal surgery of the pituitary adenoma with preoperative administration of first-generation long-acting somatostatin analogues (SSA; lanreotide and/or octreotide; for at least 3 months), and the other 21 patients received only pharmacotherapy with SSA. Lanreotide Autogel was administered 4-weekly by deep subcutaneous injections of 120 mg, and octreotide LAR was applied by intramuscular injections of 20 mg or 30 mg every 28 days. Due to the lack of follow-up data, four patients from both two groups were excluded from the further analysis (*n* = 3 vs. *n* = 1, respectively). A total of 58 patients with acromegaly were re-evaluated by clinical and laboratory assessment a minimum of two months after surgery or only SSA therapy [42,43].

Acromegaly was considered cured after surgery when both the IGF-1 level was within the normal age and gender-adjusted range and the GH level was <1 ng/mL during OGTT [38]. Simultaneously, it was defined as controlled during only pharmacological treatment if both the circulating IGF-1 level was within the normal age and gender-adjusted ranges and random GH was <1 ng/mL [38]. Taking into account the above-mentioned criteria, patients with acromegaly were next classified into four groups as cured (*n* = 23)/controlled (*n* = 5) and uncontrolled (after surgery: *n* = 15; after pharmacotherapy: *n* = 15) during follow-up.

### 2.3. Clinical, Radiological and Laboratory Assessments

Patients’ demographic data, such as age, sex, and body mass index (BMI) were calculated using the typical formula: weight (in kilograms) divided by height (in meters) squared were collected. The clinical course of acromegaly, including duration of the disease prior to diagnosis, typical signs and symptoms, cardiovascular complications, glucose homeostasis disorders, and other comorbidities were retrieved from the medical records and characterized. The pituitary adenoma mass effects, such as headache and visual impairment were evaluated both in acromegaly and NFPA groups.

MRI with contrast enhancement of the sellar region was performed before and after treatment in patients with acromegaly and at diagnosis of NFPAs. The tumor maximum diameter (MD) of coronal, sagittal, and axial planes was measured, and the tumor was categorized as microadenoma (<10 mm), macroadenoma (≥10 mm), or giant pituitary adenoma (>40 mm). Moreover, sphenoid or cavernous sinus invasion and compression of the optic chiasm were evaluated [44]. All patients from the study groups underwent visual field testing by an ophthalmologist.

All measurements were performed in a centralized laboratory and blood samples were taken at 8 a.m. The plasma GH concentration was measured using the electrochemiluminescence method with the Cobas^®^ 6000 modular analyzer (Roche Diagnostics, Mannheim, Germany; from 2009 to 2020) or by the immunoradiometric assay (IRMA) method (Immunotech, Prague, Czech Republic; from 2005 to 2009) with a detection limit of 0.03 ng/mL (WHO International Standard 98/574). The IGF-1 concentration was determined by the electrochemiluminescence method with the Cobas^®^ 8000 modular analyzer (Roche Diagnostics, Mannheim, Germany; from 2009 to 2020, WHO International Standard 02/254, detection limit of 7.0 ng/mL) or by the radioimmunoassay (RIA) method (from 2005 to 2009; WHO International Standard 87/518, detection limit 3.4 ng/mL). Normalized IGF-1 was expressed as the IGF-1 upper limit of normal (ULN) range for age and sex [45]. The functioning of other pituitary axes was evaluated by analyzing plasma levels of thyroid-stimulating hormone (TSH), free thyroxine, free triiodothyronine, adrenocorticotropic hormone (ACTH), the daily rhythm of cortisol, follicle-stimulating hormone (FSH), luteinizing hormone (LH), estradiol (females only), testosterone (males only), and prolactin (PRL). Hypopituitarism was defined as the presence of at least one pituitary deficiency documented biochemically through basal pituitary function tests, and when necessary dynamic tests were performed following the Endocrine Society Guidelines [46]. To exclude hormonal hypersecretion of the pituitary adenoma, additional functional tests were performed, including screening for glucocorticoid excess with an overnight dexamethasone suppression test, assessing for macroprolactin and metoclopramide with a test in line with recommendations to rule out functional hyperprolactinemia and prolactin-secreting pituitary adenoma if necessary [47,48]. Based on literature data, PRL concentration < 100 mg/mL usually indicates stalk dysfunction and is allowed to exclude an autonomous secretion of PRL in patients with acromegaly and NFPAs [41,49].

Moreover, patients with acromegaly were classified according to the glucose status as those with normal glucose homeostasis, pre-diabetes (impaired fasting glucose, IFG; impaired glucose tolerance, IGT), and diabetes mellitus (DM) type 2/secondary form of diabetes mellitus. IFG was defined as the fasting plasma glucose (FPG) concentration within 100–125 mg/dL, IGT as plasma glucose within 140–199 mg/dL at 120 min of OGTT, and DM according to current recommendations or the use of anti-diabetic drugs [50]. However, OGTT was not performed as a part of the routine evaluation of the NFPA and control groups. It was performed only in patients with elevated FPG in the upper limit of normal range or the presence of risk factors for the development of diabetes mellitus in the NFPA and control groups [50]. Similarly, glycated hemoglobin (HbA1c) values were not routinely used in the assessment of patients’ blood glucose control in both the NFPA and control groups in our retrospective study [50].

The CBC data obtained from venous blood samples of all participants from the study and control groups, determined by an automated hematology analyzer using the fluorescence flow cytometry method, were analyzed. In case of concomitant secondary adrenal insufficiency at diagnosis, the CBC tests were acquired before the implementation of hydrocortisone replacement therapy. The selected parameters of the WBC system and its subpopulations: neutrophil, lymphocyte, monocyte counts as well as platelet (PLT) counts and mean platelet volume (MPV) were measured. Taking into account these retrospectively available biochemical data, the following scores were calculated: NLR, PLR values by dividing the absolute neutrophil and platelet counts by the absolute lymphocyte count, respectively, and LMR by dividing the absolute lymphocyte count by the absolute monocyte count. Due to the missing data in paper-based charts, the MPV and MPV/PLT ratios were evaluated in 40 patients with acromegaly, 119 of those with NFPAs, and in all subjects from the controls. Additionally, the systemic immune-inflammation index (SII) was obtained in all participants by multiplying the absolute PLT count and NLR [17].

### 2.4. Statistical Analysis

Non-parametric tests were used for the statistical analysis due to the non-normal distribution of the most examined variables and the small sample size of the study group. Data are presented as medians with interquartile ranges (IQRs) for continuous variables independently of the Gaussian distribution. Categorical ones are expressed as numbers with percentages (%). The Chi-Square test was performed to determine sex distribution between groups. The Wilcoxon rank-sum test was used to compare paired continuous variables. The comparison of the values of CBC-derived indicators dependent on selected variables was carried out using non-parametric tests: the Kruskal-Wallis one-way analysis of variance by ranks (for more than two groups) or the U-Mann-Whitney (for two groups). The Spearman’s correlation analysis was used to calculate the correlation coefficients. The area under the curve (AUC) value using receiver operating characteristic (ROC) analysis was performed to evaluate the optimal cut-off value for selected parameters. Two-way ANOVA-type nonparametric analysis for Longitudinal Data (nparLD) was carried out to analyze the interaction of time-points and treatment groups as simultaneous influence factors on variables. All *p*-values are two-tailed, and *p*-values < 0.05 were considered as statistically significant. The statistical analysis was performed with STATISTICA 13.3.0. software for Windows (TIBCO Software Inc., Palo Alto, CA, USA) and R Studio version 3.6.1 (R Studio, Boston, MA, USA).

## 3. Results

### 3.1. Baseline Characteristics of Study and Control Groups

The median age at diagnosis and gender distribution were not statistically different between patients with acromegaly and NFPAs (*p* > 0.05). In terms of age, significant differences were detected only between acromegaly and controls (*p* = 0.006), and NFPAs and controls (*p* = 0.012). The majority (*n* = 131; 66.8%) of individuals with pituitary adenomas were females. BMI was significantly higher in patients with acromegaly compared with NFPAs (*p* = 0.001) and controls (*p* < 0.001). The baseline demographic and clinical characteristics of patients with pituitary adenomas and controls are shown in Table 1.

Macroadenoma was found in 72.6% of patients with acromegaly and 56.7% of subjects with NFPAs (*p* = 0.034). However, there were no statistically significant differences in the initial adenoma MD between both studied groups (*p* = 0.241). No significant differences were found in terms of the presence of at least one feature of the tumor’s invasiveness and pituitary deficiency in acromegaly and NFPAs (56.5% vs. 51.5%; *p* = 0.520 and 27.4% vs. 35.1%; *p* = 0.176, respectively). Nevertheless, secondary hypothyroidism and adrenal insufficiency were noted significantly higher in NFPAs (*p* = 0.002 and *p* = 0.009, respectively) than in GH-secreting adenomas, probably as a consequence of exclusion criteria of the study (Table 1).

Glucose homeostasis disorders were noted more frequently in acromegaly than in NFPA patients (*p* < 0.05). At diagnosis of acromegaly, 21.0% (*n* = 13) of individuals had DM, 48.4% (*n* = 30) had pre-DM, and only 30.6% (*n* = 19) were defined as normoglycemic subjects (Table 1). Hypertension was observed significantly more often in acromegaly than in NFPAs (*p* < 0.001). However, there were no significant differences in the incidence of other concomitant diseases, such as coronary artery disease, cardiac arrhythmias, and obstructive sleep apnea syndrome (all *p* > 0.05). Acromegaly patients had carpal tunnel syndrome more often than individuals with NFPAs (*p* = 0.002) (Table 1).

It was observed that patients with acromegaly treated with SSA were older than those who underwent surgery (60 vs. 53; *p* = 0.026). The median initial adenoma MD was 12.5 mm (IQR 8.1–17.5) in the non-surgery group and 14.5 mm (IQR 10.0–25.0) in individuals from surgery one (*p* = 0.242). Lanreotide Autogel and octreotide LAR were used with the same frequency (45% vs. 45%), and only two patients received switching SSA therapy. Furthermore, all patients who underwent transsphenoidal resection of the pituitary tumor were treated with SSA as a preoperative preparation (lanreotide Autogel: 36.8%, *n* = 14, octreotide LAR: 52.6%, *n* = 20 or octreotide LAR treatment switched to lanreotide Autogel: 10.5%, *n* = 4). The characteristics of patients with acromegaly according to treatment methods are shown in Table S1.

Regarding hematological parameters, the significant intergroup differences in median values of WBC, neutrophil, lymphocyte, NLR, LMR, PLR, and SII were revealed in the Kruskal-Wallis one-way analysis of variance by ranks test. The NLR, PLR, SII values, and neutrophil count were significantly higher in acromegaly than in NFPAs (all *p* ≤ 0.001), whereas lymphocyte count turned out to be statistically lower in Group 1 as compared with Group 2 (*p* = 0.001). Moreover, no statistically significant differences between NFPAs and controls were observed in the case of CBC-derived parameters, except for the statistically lower value of the lymphocyte count (*p* = 0.023). The comparisons of hematological and other laboratory parameters, and the above-mentioned ratios among patients with acromegaly, NFPA subjects and controls are presented in Table 2.

There were no statistically significant differences in analyzed CBC-derived parameters according to glucose homeostasis disturbances in patients with acromegaly. Subjects with acromegaly and coexisting hypertension presented lower platelet and lymphocyte counts (1.57, IQR 1.37–1.87 vs. 2.04, IQR 1.67–2.24; 216.0, IQR 200.0–273.0 vs. 276.0, IQR 221.0–319.0, respectively; both *p* = 0.012). However, other analyzed hematological indices did not differ between these two groups (*p* > 0.05). Moreover, there were no significant differences in CBC-derived parameters and ratios in patients with GH-secreting tumors concerning coronary artery disease, obstructive sleep apnea, carpal tunnel syndrome, degenerative changes in bones and joints, and benign neoplasms (all *p* > 0.05). All concomitant diseases were stable at the time of assessment.

### 3.2. Hematological Parameters Reflecting Inflammatory Process: Comparison with Traditional Markers of Acromegaly

To further investigate the potential predictive accuracy of selected CBC-derived parameters detecting acromegaly from NFPAs, an ROC curve analysis was performed. As illustrated in Table 3, all analyzed CBC-derived indices displayed a very low predictive value for acromegaly with AUC ranging from 0.60 to 0.78. Only, the IGF-1 upper limit of normal range, and the random IGF-1 and GH value showed the best accuracy distinguishing between acromegaly and NFPAs (AUC = 1.00, 100% sensitivity and specificity, AUC = 0.98, 92% sensitivity and 96% specificity, and AUC = 0.97, 92% sensitivity and 89% specificity, respectively; for all *p* < 0.001). The cut-off value of IGF-1 upper limit of the normal range was 0.99.

### 3.3. Correlation between Selected Clinical, Radiological and Laboratory Data in Acromegaly and NFPAs

The study revealed that there are significant positive correlations between neutrophil count, NLR, PLR, SII values and random GH (r = 0.207, *p* = 0.004; r = 0.460, *p* < 0.001; r = 0.326, *p* < 0.001; r = 0.390, *p* < 0.001, respectively) and IGF-1 normalized to ULN range for age and sex (r = 0.273, r = 0.502, r = 0.338, r = 0.466, all *p* ≤ 0.001, respectively) in all patients with GH-secreting tumors and NFPAs when these ones were analyzed together. Moreover, negative correlations were revealed in case of lymphocyte count, LMR, and random GH (r = −0.344, *p* < 0.001; r = −0.192, *p* = 0.007, respectively) and IGF-1 normalized to ULN range for age and sex (r = −0.318, *p* < 0.001; r = −0.152, *p* = 0.034, respectively) in the whole group involving acromegaly and NFPA patients. Similar significant positive correlations were also investigated between NLR, PLR, SII values and GH (r = 0.282, *p* < 0.001 vs. r = 0.180, *p* = 0.037 vs. r = 0.177, *p* = 0.041, respectively) and IGF-1 (r = 0.182, *p* = 0.036 vs. r = 0.270, *p* = 0.002 vs. r = 0.239, *p* = 0.005, respectively) concentrations in NFPAs, whereas such relationships were not observed in patients with acromegaly. Among all patients with pituitary tumors, none of the correlations between CBC-derived ratios, GH/IGH-1 and adenoma MD was noted. Moreover, there were no significant correlations between the duration of acromegaly symptoms prior to diagnosis and the age at diagnosis along with analyzed indices in both studied groups, except for negative correlation between PLT count and duration of symptoms in acromegaly (r = −0.261, *p* = 0.041).

### 3.4. Changes in Systemic Inflammatory Parameters during Follow-up in Patients with Acromegaly

**Figure 2:**
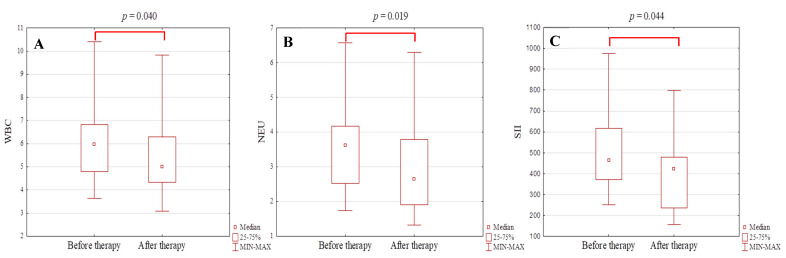
(**A**–**C**) Differences in hematological parameters and derived ratios in patients with acromegaly during pretreatment period and after SSA therapy (*n* = 20). The Wilcoxon rank-sum test was used in the analysis. *p*-value < 0.05 was statistically significant. WBC—white blood cell; NEU—neutrophil; SII—systemic immune-inflammation index.

To analyze the effect of the treatment method on changes in CBC-derived inflammatory markers, patients were categorized into surgery and non-surgery groups. The median follow-up was 10.0 (IQR 2.3–17.8) months in the case of the SSA group and 8.8 (IQR 7.3–10.8) months in the surgery one (with median 2.8 months, IQR 2.0–5.0 counting from the date of operation). There were no significant differences in the initial hematological indices between both surgery and SSA groups. In the present study, no statistically significant differences in preoperative and postoperative hematological parameters and derived ratios in patients with acromegaly were noted. However, significant differences in WBC (5.98, IQR 4.79–6.84 vs. 5.00, IQR 4.33–6.30; *p* = 0.040), neutrophil (3.63, IQR 2.53–4.17 vs. 2.66, IQR 1.91–3.78; *p* = 0.019) counts, and SII (464.40, IQR 372.40–616.70 vs. 422.84, IQR 235.84–477.74; *p* = 0.044) value were displayed in patients with acromegaly before and after SSA therapy (as shown in Figure 2A–C).

To investigate the differences in the initial values of CBC parameters and calculated ratios concerning the effectiveness of treatment, patients from both the surgery and SSA groups were divided into two groups according to response to treatment. Patients who failed to achieve cure with surgery had significantly higher initial NLR, PLR, and SII values compared with cured ones (1.95 vs. 1.50; *p* = 0.044, 140.74 vs. 114.43; *p* = 0.048, and 513.53 vs. 369.41; *p* = 0.035, respectively) (Table 4). The incidence of concomitant diseases in patients cured with surgery was similar (all *p* > 0.05) to individuals who were uncontrolled after operation as follows: hypertension 60.9% (*n* = 14) vs. 66.7% (*n* = 10), glucose homeostasis disturbances 73.9% (*n* = 17) vs. 66.7% (*n* = 10), coronary artery disease 13.0% (*n* = 3) vs. 13.3 (*n* = 2). Moreover, there were no significant differences in the initial inflammatory CBC-derived indices concerning the above-mentioned comorbidities and cured/uncontrolled acromegaly after surgery (*p* > 0.05). No statistically significant differences were noted in baseline hematological parameters in patients with acromegaly who received only SSA therapy concerning treatment efficacy (all *p* > 0.05).

**Figure 3:**
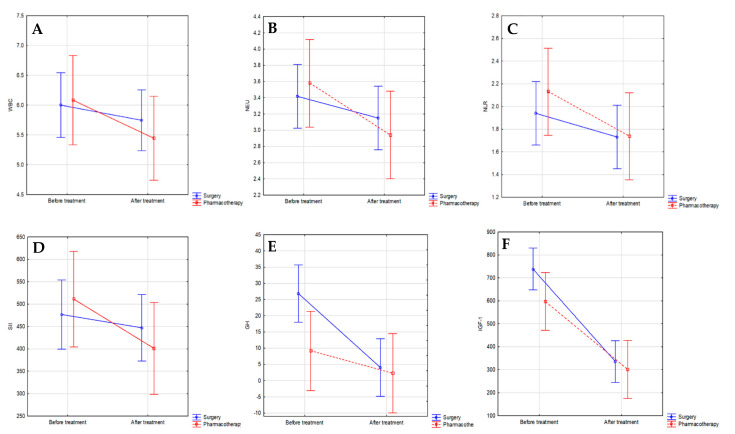
(**A**–**F**) Analysis using nparLD resulting from the effect of different factors (methods of treatment: pharmacotherapy, surgery; time: before and after therapy) on blood count parameters. (**A**) The interaction effect of the time (*p* = 0.022). WBC before: surgery 5.67 (IQR 4.80–7.15), pharmacotherapy 5.98 (IQR 4.79–6.84); WBC after: surgery 5.56 (IQR 4.73–6.64), pharmacotherapy 5.00 (IQR 4.33–6.30). (**B**) The interaction effect of the time (*p* = 0.007). NEU before: surgery 3.16 (IQR 2.44–4.05), pharmacotherapy 3.63 (IQR 2.53–4.17); NEU after: surgery 2.89 (IQR 2.41–3.73), pharmacotherapy 2.66 (IQR 1.91–3.78). (**C**) The interaction effect of the time (*p* = 0.008). NLR before: surgery 1.75 (IQR 1.36–2.32), pharmacotherapy 1.98 (IQR 1.30–2.85); NLR after: surgery 1.57 (IQR 1.22–2.14), pharmacotherapy 1.56 (IQR 1.06–2.21). (**D**) The interaction effect of the time (*p* = 0.012). SII before: surgery 417.46 (IQR 313.21–599.79), pharmacotherapy 464.40 (IQR 372.40–616.70); SII after: surgery 380.76 (IQR 268.08–561.65), pharmacotherapy 422.84 (IQR 235.84–477.74). (**E**) The interaction effect of the group and the time (*p* = 0.004). GH before: surgery 11.65 (IQR 3.80–25.10), pharmacotherapy 4.61 (IQR 2.31–9.41); GH after: surgery 0.87 (IQR 0.50–3.59), pharmacotherapy 1.56 (IQR 0.64–2.78). (**F**) The interaction effect of the group and the time (*p* = 0.012). IGF-1 before: surgery 675.00 (IQR 565.00–900.00), pharmacotherapy 534.50 (IQR 380.95–759.50); IGF-1 after: surgery 242.35 (IQR 177.00–357.60), pharmacotherapy 277.60 (IQR 198.85–393.70). *p*-value < 0.05 was statistically significant. WBC—white blood cell; NEU—neutrophil; NLR—neutrophil-to-lymphocyte ratio; SII—systemic immune-inflammation index; GH—growth hormone; IGF-1—insulin-like growth factor 1.

The analysis using nparLD displayed the significant effect of time (before vs. after treatment) on WBC (*p* = 0.020), neutrophil (*p* = 0.007) counts, and NLR (*p* = 0.008), SII (*p* = 0.012) values in all acromegaly patients (Table S2). The significant effect of the group, time considered separately, as well as the interaction between the group and the time simultaneously were recorded in GH (*p* = 0.034, *p* < 0.001, *p* = 0.004, respectively), IGF-1 (*p* = 0.027, *p* < 0.001, *p* = 0.012, respectively) concentrations, and adenoma MD (*p* = 0.042, *p* < 0.001, *p* < 0.001, respectively) (Figure 3A–F).

## 4. Discussion

To the best of our knowledge, this study is the first to comprehensively assess hematological parameters of systemic inflammation in patients with GH-secreting pituitary adenomas as compared with NFPAs and controls, particularly with respect to changes of these markers during follow-up. Our findings demonstrated that NLR, PLR, SII values, and neutrophil count were significantly higher in patients with acromegaly compared with the NFPA group, while lymphocyte count turned out to be statistically lower in acromegaly patients.

The exact mechanisms underlying the development of chronic subclinical inflammation in the course of GH-secreting pituitary adenomas still remain unclear [5,10]. Previous in vitro studies demonstrated that the direct effects of GH are probably mediated by GH-specific receptors located in the membrane of GH-responsive cells [51]. Literature data suggest that IGF-1 exerts a proinflammatory effect even better than GH [52]. An additional argument confirming the inflammatory background of acromegaly is the fact that IGF-1 attenuates the production of monocyte-derived proinflammatory cytokines, including tumor necrosis factor α (TNF-α), interleukin 6 (IL-6), interleukin 8 (IL-8), interleukin 1 β (IL-1 β), via mitogen-activated protein kinase (MAPK). Moreover, IGF-1 increases interferon γ (IFN-γ), interleukin 17 (IL-17), and interleukin 22 (IL-22) release by microbial Toll-like Receptor (TLR) ligands. Other findings proved that GH and IGF-1 may increase neutrophil production via the granulocyte colony-stimulating factor (GCS-F) [53]. The lower lymphocyte count in peripheral blood samples from patients with acromegaly might be the result of lymphocytes migration and infiltration of the tumor microenvironment [54,55]. Some authors suggest that the infiltration of CD4+ and CD8+ T-cells is closely correlated with higher GH levels and GH-secreting adenomas exhibited significantly more T-cells than non-GH pituitary adenomas [54]. This hypothesis partially explained neutrophilia and lymphocytopenia and increased the NLR value in the course of acromegaly which was observed in our study [5,8]. However, some authors reported no significant differences between blood cell-derived parameters and ratios between GH-secreting tumors and other pituitary adenoma subtypes [32]. It is worth noticing that NFPA patients who suffered apoplexy were included in that study which could be associated with the exaggerated WBC, neutrophil and monocyte counts, and decreased platelet count [32].

SII is a novel integrated marker of systemic inflammation and it results from a combination of NLR and PLT [17]. It has already been extensively studied as a marker of prognosis and mortality of patients with coronary artery disease and solid tumors [17]. Until now, there is only a single study concerning the relationship between SII and pituitary adenomas [32]. In accordance with previous data, we proved that the median platelet count was the lowest in patients with acromegaly compared with NFPAs and controls, but no statistical intergroup differences were observed [35]. It is well established that acromegaly is associated with increased morbidity and mortality due to cardiovascular diseases [5]. Notwithstanding, there is still a lack of consensus whether it is related to conventional risk factors, chronic subclinical inflammation or it is a result of the direct impact of GH/IGF-1 excess on cardiovascular function [36]. The multicenter, retrospective cohort study indicated that platelet counts are prone to alterations in the natural course of different cancers [56]. Thrombocytopenia may occur as a result of thrombopoiesis impairment by cancer-induced bone marrow suppression, platelet consumption, and cancer-induced platelet aggregation [32,56]. The higher PLR value in acromegaly than in NFPAs and controls in our research may be a result of the above-mentioned changes in lymphocytes and no significant differences in platelet counts. Although, we failed to find any distinctions in MPV and MPV/PLT values between the three analyzed groups. These results are in contrast with those obtained by Arpaci et al., Demirpence et al., and Unübol et al., who noted significantly higher MPV value in acromegaly compared to healthy individuals [34,35,37].

In the current study, correlations between CBC-derived parameters and selected clinical and radiological variables were analyzed. The study revealed that there are significant positive correlations between neutrophil count, NLR, PLR, SII values and random GH, and IGF-1 ULN for age and sex in all patients with GH-secreting tumors and NFPAs when these ones were analyzed together. We did not observe any correlations between hematological parameters and GH, IGF-1 concentrations in patients with acromegaly when analyzed as a single group which may result from the small sample size. This partially stands in agreement with the research conducted by Marques et al. who showed only the negative correlation between IGF-1 and PLT [32]. Üçler et al., in a retrospective analysis of data from 61 patients with acromegaly, reported significant positive correlations between IGF-1 concentration and both NLR and PLR, respectively [33]. However, the authors found no statistically significant differences in NLR and PLR values among patients with acromegaly according to glucose status [33], similarly to our results. On the other hand, significant positive correlations between NLR, PLR, SII and GH, and IGF-1 were noted in the NFPA group during our investigation. Taking into consideration conflicting data about the link between tumor size and inflammatory markers, we performed the analysis of the correlation between CBC-derived indices and adenoma MD in GH-secreting tumors and NFPAs, but no correlations were observed [57,58,59]. Moreover, we did not observe any significant differences in the calculated ratios in acromegaly patients concerning glucose metabolism disturbances, hypertension, coronary artery disease, obstructive sleep apnea, carpal tunnel syndrome, degenerative changes in bones and joints, and benign neoplasms.

Some patients diagnosed with pituitary tumor may present only subtle signs and symptoms of acromegaly or may have no symptoms of the disease at all [60]. Hence, distinguishing individuals with silent acromegaly from those with NFPAs is still a challenge in clinical practice [48]. An interesting finding of our work is the disclosure of no statistical differences between the NFPA group and controls. Although NLR, PLR, and SII values were significantly higher in acromegaly than in NFPAs, these inflammatory indices cannot be used to discriminate GH-secreting pituitary tumors from NFPAs due to very low diagnostic accuracy. A ROC curve analysis confirmed that traditional markers of acromegaly, such as GH/IGF-1 showed the best sensitivity and specificity ranged from 92–100% and 89–100% respectively, which is consistent with literature data [4].

Our study provides new evidence regarding changes in systemic inflammation-based markers during follow-up. Recent data focused only on differences in the MPV value and platelet count before and after therapy of patients with acromegaly. However, the results of these studies are not consistent [35,36]. Demirpence et al. revealed that the MPV value was decreased after effective surgical resection of GH-secreting pituitary adenoma, but similar changes were not observed in the case of adjuvant SSA therapy after surgery and inactive acromegaly [35]. Another study displayed no significant differences in the MPV value before and after SSA therapy as well as, surprisingly, a higher MPV value in patients with acromegaly treated by transsphenoidal removal of the tumor in the inactive period of the disease compared to the active one [36]. Following previous data, we proved that there were no statistically significant differences in the MPV and MPV/PLT values before and after therapy, regardless of the treatment method. We discovered significant differences in WBC, neutrophil counts, and SII value before and after SSA therapy. The above-mentioned changes were observed also in the preoperative and postoperative periods, but with no statistical significance. Wolters et al. discovered that acromegaly patients are characterized by a proinflammatory phenotype, especially those with uncontrolled disease [61]. Taking into consideration the above results, we examined whether there is a relationship between the initial level of CBC-derived parameters in the context of the effectiveness of treatment. It occurred that patients cured with surgical resection of the tumor presented significantly lower baseline NLR, PLR, and SII values as compared with ones who failed to achieve cure. These findings may be a clue for clinicians to evaluate the activity of inflammation after surgery, but they should be validated in large cohort studies. Moreover, we provided the analysis taking into account both the pretreatment and posttreatment assessment and the effect of the treatment method on the changes of hematological indices in acromegaly patients. Despite the significant effect of time (before vs. after treatment) on WBC, neutrophil counts, and NLR, SII values, we found that GH, IGF-1 concentrations, and adenoma MD showed statistically significant effects of group and time simultaneously. Considering the fact that this is the first study that assesses the changes of all CBC-derived parameters and ratios in patients with acromegaly before and after treatment, further research is needed to discuss these results accurately.

Notwithstanding, we would like to highlight that NLR, PLR, and SII as readily available, affordable biomarkers, are interesting for cognition of pituitary tumors microenvironment and long-lasting systemic inflammatory imprint. These indices, however, should be interpreted cautiously in a clinical context.

## 5. Limitations of the Study

This cross-sectional study has some limitations, mainly due to the retrospective nature of the research. Firstly, the study was not designed to discover the potential links between the changes in CBC-derived parameters values and other well-established, but not routinely assessed inflammatory markers, such as highly sensitive C-reactive protein (hs-CRP), erythrocyte sedimentation rate (ESR), TNF-α, IFN-γ, IL-1β, IL-6, IL-8, vascular cell adhesion molecule 1 (VCAM-1), and intercellular adhesion molecule 1 (ICAM-1). Secondly, patients with acromegaly received various doses of SSA depending on individual tolerance and the clinical course of the disease. Another limitation was the inability to perform follow-up evaluation at the same time interval and to eliminate the preoperative SSA therapy effects as potential confounders. Therefore, prospective large-scale studies in more patients with acromegaly are necessary to validate obtained findings.

## 6. Conclusions

The results of our study indicate that CBC-derived parameters differ significantly in patients with acromegaly as compared with NFPAs and healthy individuals. Although NLR, PLR, and SII values were significantly higher in acromegaly than in NFPAs, these inflammatory indices cannot be used to discriminate GH-secreting pituitary tumors from NFPAs. CBC-derived inflammatory ratios did not differ in patients with acromegaly if the occurrence of its comorbidities was taken into consideration.

The initial NLR, PLR, and SII values were observed to be significantly higher in patients with acromegaly who failed to achieve a cure with transsphenoidal pituitary removal. However, further studies to assess the temporal relationship of these changes and their correlation with other inflammatory markers are necessary to consolidate the real clinical role of these ratios.

## Figures and Tables

**Table 1:** General characteristics of the study and control groups.

Variables	Acromegaly	NFPAs	Controls	*p*-Value
	(*n* = 62)	(*n* = 134)	(*n* = 120)	
Age at diagnosis [years]	55 (44–62)	53 (36–64)	43 (26–59)	0.002 *^,1^
Female, *n* (%)	41 (66.1)	90 (67.2)	71 (59.2)	0.383 ^2^
Male, *n* (%)	21 (33.9)	44 (32.8)	49 (40.8)	
BMI [kg/m^2^] ^a^	30.3 (26.5–34.6)	27.1 (24.1–31.4)	25.1 (23.3–28.7)	<0.001 *^,1^
normal body weight, *n* (%)	10 (16.1)	46 (34.3)	60 (50.0)	<0.001 *^,1^
overweight, *n* (%)	21 (33.9)	43 (32.1)	42 (35.0)	0.885 ^1^
obesity class I, *n* (%)	18 (29.0)	33 (24.6)	15 (12.5)	0.012 *^,1^
obesity class II, *n* (%)	7 (11.3)	8 (6.0)	3 (2.5)	0.053 ^1^
obesity class III, *n* (%)	6 (9.7)	4 (3.0)	0 (0)	0.002 *^,3^
Duration of symptoms prior to diagnosis [years]	7 (2–10)	-	-	-
Random GH at diagnosis [ng/mL]	8.10 (3.59–18.50)	0.27 (0.10–0.74)	-	<0.001 *^,3^
IGF-1 at diagnosis [ng/mL]	647.8 (407.7–900.0)	136.3 (98.7–190.0)	-	<0.001 *^,3^
IGF-1 × ULN at diagnosis	3.14 (2.08–4.47)	0.63 (0.47–0.79)	-	<0.001 *^,3^
**Pituitary adenoma mass effects**				
Visual field defects, *n* (%)	8 (12.9)	33 (24.6)	-	0.110 ^3^
Headache, *n* (%)	34 (54.8)	80 (59.7)	-	0.523 ^3^
**Radiological features of pituitary adenomas**				
Primary tumour MD [mm]	14.0 (9.0–23.0)	14.0 (5.8–23.0)	-	0.241 ^3^
Microadenoma, *n* (%)	15 (24.2)	54 (40.3)	-	0.029 *^,3^
Macroadenoma, *n* (%)	45 (72.6)	76 (56.7)	-	0.034 *^,3^
Giant tumour, *n* (%)	2 (3.2)	4 (3.0)	-	**
At least 1 feature of pituitary adenoma invasiveness, *n* (%)	35 (56.5)	69 (51.5)	-	0.520 ^3^
No features of pituitary adenoma invasiveness, *n* (%)	27 (43.5)	65 (48.5)	-	0.520 ^3^
Sphenoid sinus invasion, *n* (%)	30 (48.4)	31 (23.1)	-	0.817 ^3^
Cavernous sinus invasion, *n* (%)	20 (32.3)	41 (30.6)	-	<0.001 *^,3^
Compression of the optic chiasm, *n* (%)	17 (27.4)	55 (41.0)	-	0.067 ^3^
**Hormonal status of pituitary gland**				
Secondary hypothyroidism, *n* (%)	1 (1.6)	24 (17.9)	-	0.002 *^,3^
Secondary adrenal insufficiency, *n* (%)	2 (3.2)	22 (16.4)	-	0.009 *^,3^
Hypogonadism hypogonadotropic/estrogen depletion, *n* (%)	8 (12.9)	28 (20.9)	-	0.181 ^3^
Hyperprolactinemia due to pituitary stalk deviation, *n* (%)	13 (21.0)	21 (15.7)	-	0.365 ^3^
GH deficiency, *n* (%)	-	6 (4.5)	-	**
At least 1 pituitary deficiency, *n* (%)	17 (27.4)	47 (35.1)	-	0.176 ^3^
No pituitary deficiency, *n* (%)	45 (72.6)	87 (64.9)	-	0.176 ^3^
**Glucose homeostasis disorders**				
Normoglycemia, *n* (%)	19 (30.6)	105 (78.4)	-	<0.001 *^,3^
Pre-DM, *n* (%)	30 (48.4)	17 (12.7)	-	<0.001 *^,3^
IFG, *n* (%)	18 (60)	10 (58.8)	-	<0.001 *^,3^
IGT, *n* (%)	5 (16.7)	2 (11.8)	-	0.002 *^,3^
IFG+IGT, *n* (%)	7 (23.3)	5 (29.4)	-	0.041 *^,3^
T2DM/secondary form of DM, *n* (%)	13 (21.0)	12 (9.0)	-	0.019 *^,3^
FPG [mg/dL]	105.5 (93.0–117.0)	90.0 (83.0–97.0)	90.5 (85.0–97.0)	<0.001 *^,1^
HbA1c [%]	6.2 (5.6–6.6)	5.9 (5.5–6.3)	-	0.210 ^3^
**Cardiovascular complications**				
Hypertension, *n* (%)	49 (79.0)	59 (44.0)	-	<0.001 *^,3^
Coronary artery disease, *n* (%)	10 (16.1)	12 (9.0)	-	0.141 ^3^
Cardiac arrhythmias, *n* (%)	7 (11.3)	10 (7.5)	-	0.222 ^3^
Disturbances in TTE, *n* (%)	33 (53.2)	ND ***	-	-
**Other comorbidities**				
Obstructive sleep apnea syndrome, *n* (%)	15 (24.2)	42 (31.3)	-	0.307 ^3^
Benign neoplasms, *n* (%) ^b^	28 (45.2)	ND ***	-	-
Degenerative changes in joints and bones, *n* (%)	26 (41.9)	48 (35.8)	-	0.414 ^3^
Carpal tunnel syndrome, *n* (%)	13 (21.0)	8 (6.0)	-	0.002 *^,3^

HbA1c: acromegaly *n* = 62, NFPAs *n* = 29. Values are number (%) or median (interquartile range, IQR). * *p*-value < 0.05 was statistically significant. ** this variable was detected only in isolated cases, thus the statistical analysis is impossible. *** ND—no data available (not routinely screened). ^a^ Patients were classified into four groups according to BMI value: normal body weight—BMI: 18.5–24.9 kg/m^2^, overweight—BMI: 25.0–29.9 kg/m^2^, obesity class I—BMI: 30.0–34.9 kg/m^2^, obesity class II—BMI: 35.0–39.9 kg/m^2^, obesity class III—BMI: ≥40 kg/m^2^. ^b^ Benign neoplasms were defined as the presence of colon polyps, polyp of the gallbladder, uterine fibroids, adrenal gland adenoma. ^1^ Kruskal-Wallis one-way analysis of variance by ranks test; ^2^ Chi-Square test; ^3^ U-Mann-Whitney test. NFPAs—non-functioning pituitary adenomas; MD—maximum diameter; GH—growth hormone; IGF-1—insulin-like growth factor 1; IGF-1 ULN—insulin-like growth factor 1 upper limit of normal range for age and sex; FPG—fasting plasma glucose; IFG—impaired fasting glucose; IGT—impaired glucose tolerance; DM—diabetes mellitus; T2DM—diabetes mellitus type 2; HbA1c-glycated hemoglobin; TTE—trans-thoracic echocardiography.

**Table 2:** Baseline characteristics of laboratory data among three groups.

Variables	Group 1 Acromegaly (*n* = 62)	Group 2 NFPAs (*n* = 134)	Group 3 Controls (*n* = 120)	*p*-Value	*p* (Groups 1 & 2)	*p* (Groups 1 & 3)	*p* (Groups 2 & 3)
WBC [10^9^/L]	5.83 (4.80–6.98)	5.46 (4.58–6.43)	5.66 (5.19–6.68)	0.043 *	NS	NS	0.050
NEU [10^9^/L]	3.24 (2.48–4.05)	2.68 (2.15–3.18)	2.76 (2.50–3.22)	<0.001 *	<0.001 *	0.012 *	NS
LYM [10^9^/L]	1.69 (1.45–2.16)	2.11 (1.73–2.53)	2.24 (1.96–2.60)	<0.001 *	0.001 *	<0.001 *	0.023 *
NLR	1.84 (1.36–2.61)	1.29 (1.08–1.52)	1.23 (1.10–1.41)	<0.001 *	<0.001 *	<0.001 *	NS
MONO [10^9^/L]	0.35 (0.27–0.47)	0.37 (0.30–0.43)	0.37 (0.32–0.46)	0.097	NS	NS	NS
LMR	5.15 (3.76–6.55)	5.93 (4.89–7.11)	5.83 (4.90–7.09)	0.039 *	NS	NS	NS
PLT [10^9^/L]	223.0 (204.0–289.0)	228.3 (193.0–276.0)	235.5 (194.5–276.5)	0.811	NS	NS	NS
PLR	131.15 (112.30–177.57)	109.96 (93.33–137.43)	100.86 (84.55–123.17)	<0.001 *	0.001 *	<0.001 *	NS
MPV [fL]	7.6 (7.0–8.2)	7.8 (7.3–8.5)	7.7 (7.3–8.5)	0.437	NS	NS	NS
MPV/PLT	0.034 (0.027–0.039)	0.034 (0.027–0.041)	0.032 (0.027–0.044)	0.825	NS	NS	NS
SII	439.75 (325.73–599.79)	301.07 (233.52–359.31)	291.47 (227.94–354.24)	<0.001 *	<0.001 *	<0.001 *	NS

MPV and MPV/PLT: acromegaly *n* = 40, NFPAs *n* = 119, controls *n* = 120. Other parameters were assessed in all participants.; Values are median (interquartile range, IQR). * *p*-value < 0.05 was statistically significant. Kruskal-Wallis one-way analysis of variance by ranks test was used in the analysis. NS—no significance; WBC—white blood cell; NEU—neutrophil; LYM—lymphocyte; NLR—neutrophil-to-lymphocyte ratio; MONO—monocyte; LMR—lymphocyte-to-monocyte ratio; PLT—platelet; PLR—platelet-to-lymphocyte ratio; MPV—mean platelet volume; MPV/PLT—mean platelet volume-to-platelet ratio; SII—systemic immune-inflammation index.

**Table 3:** Comparison of inflammatory markers and GH/IGF-1 in the distinction of acromegaly from NFPAs.

	AUC	SE	95% CI	Sensitivity	Specificity	Cut-Off	*p*-Value
NEU	0.68	0.04	0.60–0.77	0.42	0.91	3.60	<0.001 *
LYM	0.66	0.04	0.58–0.75	0.76	0.24	1.70	<0.001 *
NLR	0.78	0.04	0.70–0.86	0.55	0.99	1.79	<0.001 *
LMR	0.60	0.05	0.51–0.69	0.50	0.72	5.04	0.021
PLR	0.67	0.04	0.58–0.75	0.76	0.57	112.30	<0.001 *
SII	0.77	0.04	0.69–0.85	0.53	0.90	425.98	<0.001 *
Random GH	0.97	0.01	0.95–0.99	0.92	0.89	1.66	<0.001 *
IGF-1	0.98	0.01	0.96–1.00	0.92	0.96	274.00	<0.001 *
IGF-1 × ULN	1.00	0.00	1.00–1.00	1.00	1.00	0.99	<0.001 *

* *p*-value < 0.05 was statistically significant. NEU—neutrophil; LYM—lymphocyte; NLR—neutrophil-to-lymphocyte ratio; LMR—lymphocyte-to-monocyte ratio; PLR—platelet-to-lymphocyte ratio; SII—systemic immune-inflammation index; GH—growth hormone; IGF-1—insulin-like growth factor 1, IGF-1 ULN—insulin-like growth factor 1 upper limit of normal range for age and sex; AUC—Area under the curve; SE—Standard Error; CI 95%—Confidence Interval 95%.

**Table 4:** Differences in initial values of CBC parameters and calculated ratios according to treatment effectiveness in patients with acromegaly who underwent surgical removal of pituitary adenoma.

Variables	Cured with Surgery *n* = 23	Uncontrolled with Surgery *n* = 15	*p*-Value
WBC [10^9^/L]	5.57 (4.75–7.15)	5.77 (5.14–7.42)	0.637
NEU [10^9^/L]	2.92 (2.41–4.05)	3.34 (3.11–5.17)	0.095
LYM [10^9^/L]	1.87 (1.55–2.45)	1.67 (1.44–1.92)	0.153
NLR	1.50 (1.34–1.94)	1.95 (1.51–3.16)	0.044 *
MONO [10^9^/L]	0.31 (0.27–0.48)	0.30 (0.24–0.50)	0.637
LMR	5.52 (4.54–8.04)	5.54 (3.76–6.55)	0.746
PLT [10^9^/L]	212.0 (203.0–289.0)	236.0 (206.00–313.0)	0.442
PLR	114.43 (85.64–167.60)	140.74 (123.35–194.00)	0.048 *
MPV [fL]	7.5 (7.2–8.2)	7.2 (7.0–8.5)	0.459
MPV/PLT	0.03 (0.03–0.04)	0.03 (0.02–0.04)	0.187
SII	369.41 (271.91–473.54)	513.53 (355.28–777.83)	0.035 *
Random GH at diagnosis [ng/mL]	6.36 (2.70–14.60)	18.50 (5.46–67.45)	0.003 *
IGF-1 at diagnosis [ng/mL]	671.7 (576.0–878.0)	872.9 (399.0–903.0)	0.723
IGF-1 × ULN at diagnosis	3.43 (2.48–4.60)	2.94 (2.35–4.63)	0.860
adenoma MD [mm]	13 (8–22)	25 (14–28)	0.016 *

MPV and MPV/PLT: cured with surgery *n* = 13, uncontrolled with surgery *n* = 11. Other parameters were assessed in all participants.; Values are median (interquartile range, IQR). * *p*-value < 0.05 was statistically significant. U-Mann-Whitney test was used in the analysis. WBC—white blood cell; NEU—neutrophil; LYM—lymphocyte; NLR—neutrophil-to-lymphocyte ratio; MONO—monocyte; LMR—lymphocyte-to-monocyte ratio; PLR—platelet-to-lymphocyte ratio; MPV—mean platelet volume; MPV/PLT—mean platelet volume-to-platelet ratio; SII—systemic immune-inflammation index; GH—growth hormone; IGF-1—insulin-like growth factor 1; IGF-1 ULN—insulin-like growth factor 1 upper limit of normal range for age and sex; MD—maximum diameter.

## Data Availability

The source data that support the findings of this study are available from the corresponding author(s) on reasonable request.

## Supplementary Materials

The following are available online at https://www.mdpi.com/article/10.3390/jcm10173997/s1, Table S1: Baseline characteristics of patients with acromegaly according to treatment methods. Table S2: Results of the model from the nparLD R package, testing for the effects of method of treatment (surgery, pharmacotherapy) and time (before treatment, after treatment).

## Abbreviations

BMI	Body Mass Index
CBC	complete blood count
GH	growth hormone
IGF-1	insulin-like growth factor 1
LMR	lymphocyte-to-monocyte ratio
LYM	lymphocyte
MD	maximum diameter
MONO	monocyte
MPV	mean platelet value
MPV/PLT	mean platelet volume-to-platelet ratio
MRI	magnetic resonance imaging
NEU	neutrophil
NFPA	non-functioning pituitary adenoma
NLR	neutrophil-to-lymphocyte ratio
PLR	platelet-to-lymphocyte ratio
SII	systemic immune-inflammation index
SSA	somatostatin analogue
WBC	white blood cell
