# Supplementary material for: Neutrophil-to-Lymphocyte, Platelet-to-Lymphocyte Ratios, and Systemic Immune-Inflammation Index as Potential Biomarkers of Chronic Inflammation in Patients with Newly Diagnosed Acromegaly: A Single-Centre Study
Source: J Clin Med. 2021 Sep 3;10(17):3997. doi: 10.3390/jcm10173997 (PMC8432467; doi:10.3390/jcm10173997)
Supplement: Supplementary file 1 [file jcm-10-03997-s001.zip › jcm-1344814-supplementary.pdf]

Table S1. Baseline characteristics of patients with acromegaly according to treatment methods.

Variables	SSA therapy $n = 20$	Surgery $n = 38$	p-Value
Age [years]	60 (54–67)	53 (41 – 60)	0.026 * [1]
Female, n (%)	16 (80)	23 (60.5)	0.154 [2]
Male, n (%)	4 (20)	15 (39.5)	
BMI [kg/m^2]	33.3 (27.2 – 35.5)	29.0 (26.5 – 33.5)	0.127 [1]
Random GH at diagnosis [ng/mL]	4.61 (2.31 – 9.41)	11.65 (3.80 – 25.10)	0.032 * [1]
IGF-1 at diagnosis [ng/mL]	534.50 (380.95 – 759.50)	675.0 (565.0 – 900.0)	0.087 [1]
IGF-1, x ULN at diagnosis	3.49 (1.93 – 4.12)	3.41 (2.48 – 4.60)	0.213 [1]
Lanreotide Autogel, n (%)	9 (45)	-	-
Octreotide LAR, n (%)	9 (45)	-	-
Switching SSA therapy	2 (10)	-	-
Median follow-up [months]	10.0 (2.3 – 17.8)	8.8 (7.3 – 10.8)<br>2.8 (2.0 – 4.8) ***	0.852 [1]
**Pituitary adenoma mass effects**			
Visual field defects, n (%)	5 (25)	3 (7.9)	0.077 [1]
Headache, n (%)	10 (50)	21 (55.2)	0.712 [1]
**Radiological features of pituitary adenomas**			
Primary tumour MD [mm]	12.5 (8.1 – 17.5)	14.5 (10.0 – 25.0)	0.242 [1]
Microadenoma, n (%)	6 (30)	8 (21.1)	0.383 [1]
Macroadenoma, n (%)	14 (70)	29 (76.3)	0.307 [1]
Giant tumour, n (%)	0 (0)	1 (2.6)	**
At least 1 feature of pituitary adenoma invasiveness, n (%)	8 (40)	25 (65.8)	0.063 [1]
No features of pituitary adenoma invasiveness, n (%)	12 (60)	13 (34.2)	
Sphenoid sinus invasion, n (%)	6 (30)	22 (57.9)	0.046 * [1]
Cavernous sinus invasion, n (%)	3 (15)	15 (39.5)	0.059 [1]
Compression of the optic chiasm, n (%)	6 (30)	11 (29.0)	0.942 [1]
**Hormonal status of pituitary gland**			
Secondary hypothyroidism, n (%)	1 (5)	0 (0.0)	-
Secondary adrenal insufficiency, n (%)	1 (5)	1 (2.6)	0.660 [1]
Hypogonadism hypogonadotropic/estrogen depletion, n (%)	3 (15)	5 (13.2)	0.859 [1]
Hyperprolactinemia due to pituitary stalk deviation, n (%)	3 (15)	9 (23.7)	0.449 [1]
At least 1 pituitary deficiency, n (%)	3 (15)	13 (34.2)	0.126 [1]
No pituitary deficiency, n (%)	17 (85)	25 (65.8)	
**Glucose homeostasis disorders**			
Normoglycemia, n (%)	6 (30)	11 (29.0)	0.942 [1]
Pre-DM, n (%)	10 (50)	19 (50.0)	0.992 [1]
IFG, n (%)	7 (35)	17 (44.7)	0.484 [1]
IGT, n (%)	6 (30)	6 (15.8)	0.212 [1]
IFG+IGT, n (%)	3 (15)	4 (10.5)	0.632 [1]
T2DM/secondary form of DM, n (%)	4 (20)	8 (21.1)	0.935 [1]
FPG [mg/dL]	108.0 (95.5 – 118)	104.0 (93.0 – 114.0)	0.529 [1]
**Cardiovascular complications**			
Hypertension, n (%)	15 (75)	24 (63.2)	0.371 [1]
Coronary artery disease, n (%)	3 (15)	5 (13.2)	0.859 [1]
Cardiac arrhythmias, n (%)	6 (30)	2 (5.3)	0.010 * [1]
Disturbances in TTE, n (%)	9 (45)	21 (55.3)	0.467 [1]
**Other comorbidities**			
Obstructive sleep apnea syndrome, n (%)	3 (15)	10 (26.3)	0.336 [1]
Benign neoplasms, n (%) [a]	10 (50)	16 (42.1)	0.575 [1]
Degenerative changes in joints and bones, n (%)	11 (55)	13 (34.2)	0.132 [1]

Carpal tunnel syndrome, *n* (%)	6 (30)	7 (18.4)	0.325 [1]

Values are number (%) or median (interquartile range, IQR). * *p*-value < 0.05 was statistically significant. ** This variable was detected only in single case, thus the statistical analysis is impossible. *** Median time of assessment after surgery. [1] U-Mann-Whitney test and [2] Chi-Square test were applied. GH – growth hormone; IGF-1 – insulin-like growth factor 1; IGF-1 ULN – insulin-like growth factor 1 upper limit of normal range for age and sex; SSA – somatostatin analogue; MD – maximum diameter; FPG – fasting plasma glucose; IFG – impaired fasting glucose; IGT – impaired glucose tolerance; DM – diabetes mellitus; T2DM – diabetes mellitus type 2; TTE – trans-thoracic echocardiography. [a] Benign neoplasms were defined as the presence of colon polyps, polyp of the gallbladder, uterine fibroids, adrenal gland adenoma.

Table S2. Results of the model from the nparLD R package, testing for the effects of method of treatment (surgery, pharmacotherapy) and time (before treatment, after treatment).

Factor	Statistic	df	*p*-Value
**WBC**			
Method of treatment	0.219	1	0.640
Time	5.272	1	0.022 *
Method of treatment x Time	1.159	1	0.282
**NEU**			
Method of treatment	0.053	1	0.817
Time	7.235	1	0.007 *
Method of treatment x Time	1.701	1	0.192
**LYM**			
Method of treatment	0.442	1	0.506
Time	0.459	1	0.498
Method of treatment x Time	0.090	1	0.765
**NLR**			
Method of treatment	0.139	1	0.709
Time	7.000	1	0.008 *
Method of treatment x Time	1.086	1	0.297
**MONO**			
Method of treatment	0.447	1	0.504
Time	2.023	1	0.155
Method of treatment x Time	0.918	1	0.338
**LMR**			
Method of treatment	1.088	1	0.297
Time	1.017	1	0.313
Method of treatment x Time	0.115	1	0.735
**PLT**			
Method of treatment	0.094	1	0.759
Time	0.371	1	0.542
Method of treatment x Time	2.419	1	0.120
**PLR**			
Method of treatment	0.579	1	0.447
Time	0.503	1	0.478
Method of treatment x Time	0.914	1	0.339
**MPV**			
Method of treatment	0.013	1	0.909
Time	1.636	1	0.201
Method of treatment x Time	0.015	1	0.902
**MPV/PLT**			
Method of treatment	0.103	1	0.749
Time	0.377	1	0.539
Method of treatment x Time	0.468	1	0.494
**SII**			
Method of treatment	0.198	1	0.657
Time	6.240	1	0.012 *
Method of treatment x Time	1.454	1	0.228
**GH**			
Method of treatment	0.924	1	0.034 *

Time	121.512	1	< 0.001 *
Method of treatment x Time	8.204	1	0.004 *
**IGF-1**			
Method of treatment	1.238	1	0.027 *
Time	94.054	1	< 0.001 *
Method of treatment x Time	2.397	1	0.012 *
**Adenoma MD**			
Method of treatment	4.048	1	0.042 *
Time	78.465	1	< 0.001 *
Method of treatment x Time	32.060	1	< 0.001 *

A total of 58 patients were enrolled in the analysis. * p-value < 0.05 was statistically significant. WBC – white blood cell; NEU – neutrophil; LYM – lymphocyte; NLR – neutrophil-to-lymphocyte ratio; MONO – monocyte; LMR – lymphocyte-to-monocyte ratio; PLT – platelet; PLR – platelet-to-lymphocyte ratio; MPV – mean platelet volume; MPV/PLT – mean platelet volume-to-platelet ratio; SII – systemic immune-inflammation index; GH – growth hormone; IGF-1 – insulin-like growth factor – 1; MD – maximum diameter.
